# Preventive Effect of Residential Green Space on Infantile Atopic Dermatitis Associated with Prenatal Air Pollution Exposure

**DOI:** 10.3390/ijerph15010102

**Published:** 2018-01-09

**Authors:** Ji-Young Lee, Dirga Kumar Lamichhane, Myeongjee Lee, Shinhee Ye, Jung-Hyun Kwon, Myung-Sook Park, Hwan-Cheol Kim, Jong-Han Leem, Yun-Chul Hong, Yangho Kim, Mina Ha, Eunhee Ha

**Affiliations:** 1 Department of Occupational and Environmental Medicine, College of Medicine, Ewha Womans University, Seoul 03760, Korea; jylee1@ewha.ac.kr (J.-Y.L.); lmj7914@gmail.com (M.L.); shinheeye@naver.com (S.Y.); 2 Department of Occupational and Environmental Medicine, School of Medicine, Inha University, Incheon 22212, Korea; dirgalamichhane@gmail.com (D.K.L.); carpediem@inha.ac.kr (H.-C.K.); ekeeper21@naver.com (J.-H.L.); 3 Department of Pediatrics, College of Medicine, Korea University, Seoul 02841, Korea; pediangel@hanmail.net; 4 Taean Environmental Health Center, Taean 32144, Korea; pms1816@gmail.com; 5 Department of Preventive Medicine, College of Medicine, Seoul National University, Seoul 02841, Korea; ychong1@snu.ac.kr; 6 Department of Occupational and Environmental Medicine, Ulsan University Hospital, College of Medicine, University of Ulsan, Ulsan 44033, Korea; yanghokm@ulsan.ac.kr; 7 Department of Preventive Medicine, College of Medicine, Dankook University, Cheonan 31116, Korea; minaha@dku.edu

**Keywords:** child health, atopic dermatitis, greenness, traffic-related air pollution, particulate matter, birth cohort

## Abstract

Few birth cohort studies have examined the role of traffic-related air pollution (TRAP) in the development of infantile atopic dermatitis (AD), but none have investigated the role of preventive factors such as green spaces. The aim of this study was to investigate whether exposure to nitrogen dioxide (NO_2_) and particulate matter with an aerodynamic diameter of <10 μm (PM_10_) during pregnancy is associated with increased risk of development of AD in 6-month-old children and also to examine how this association changes with residential green space. This study used prospective data from 659 participants of the Mothers and Children’s Environmental Health study. Subjects were geocoded to their residential addresses and matched with air pollution data modeled using land-use regression. Information on infantile AD was obtained by using a questionnaire administered to the parents or guardians of the children. The association between infantile AD and exposure to NO_2_ and PM_10_ was determined using logistic regression models. We assessed the effects of residential green spaces using stratified analyses and by entering product terms into the logistic regression models. The risk of infantile AD significantly increased with an increase in air pollution exposure during the first trimester of pregnancy. The adjusted odds ratio (OR) and 95% confidence interval (CI) were 1.219 (1.023–1.452) per 10 μg/m^3^ increase in PM_10_ and 1.353 (1.027–1.782) per 10 ppb increase in NO_2_. An increase in the green space within 200 m of residence was associated with a decreased risk of AD (OR = 0.996, 95% CI: 0.993–0.999). The stratified analysis of residential green space revealed stronger associations between infantile AD and PM_10_ and NO_2_ exposure during the first trimester in the areas in the lower tertiles of green space. This study indicated that exposure to TRAP during the first trimester of pregnancy is associated with infantile AD. Less residential green space may intensify the association between TRAP exposure and infantile AD.

## 1. Introduction

Atopic dermatitis (AD) is a chronic inflammatory skin disease that affects as many as 10–30% of children [1,2] with a typical onset during infancy. The prevalence of AD appears to have increased worldwide in recent decades [1,3]. In South Korea, the prevalence of AD in children aged 6–7 years was 5.7% and 11.2% in 2000 and 2010, respectively [4]. Despite many attempts to identify the causative factors of AD in children, they remain unknown. Although genetic factors and atopic sensitization are considered major determinants of prognosis [5], the growing incidence rate in recent years is most likely associated with environmental factors [6].

Several epidemiological studies have reported that exposure to ambient air pollutants increases the risk of AD or exacerbates AD symptoms in children [7,8]. Increasingly, exposure to air pollution during pregnancy is being considered a potential cause of AD. However, studies regarding prenatal exposure to air pollution and the development of AD are limited [9,10,11,12,13,14,15,16,17,18]. Most of these studies reported that exposures to ambient air pollutants during pregnancy increase the risk of AD in childhood. These studies differ considerably with regard to exposure assessment and outcome definitions, including the age of children. Other studies have shown no association between ambient air pollutants during pregnancy and AD in childhood [17,18].

Access to green space around homes is inversely associated with the risk of AD in children [19,20]. Green spaces have also been associated with better mental health and improved cardiovascular health in the general adult population [21,22]. Some studies have reported that residing in or near green spaces unfavorably influences allergy health outcomes in children [23,24,25]. Recent studies have suggested that the health benefits for overweight and obese children may depend on the type of green space [26], and that greenness has beneficial effects on adverse pregnancy outcomes such as low birth weight and preterm birth [27].

Inconsistent associations between air pollution exposure during pregnancy and AD have prompted further study of the relationship between air pollution, green space, and infantile AD. Although previous studies have found that infantile AD may be related to air pollution during pregnancy and that green spaces may mitigate risk, all those studies analyzed the effects of air pollution and residential greenness separately [11,13,20]. This is the first cohort study to investigate the relationship between exposure to traffic-related air pollution (TRAP), consisting of particulate matter with an aerodynamic diameter of ≤10 µm (PM_10_) and nitrogen dioxide (NO_2_), during pregnancy, with green spaces and the development of AD in children aged 6 months.

In this study, we sought to investigate whether exposure to PM_10_ and NO_2_ during different periods of pregnancy is associated with increased risk of infantile AD. Furthermore, in the stratified analysis, we investigated the combined effects of TRAP and greenness on the risk for infantile AD.

## 2. Materials and Methods

### 2.1. Study Population

This was a prospective birth cohort study conducted as a part of the Mothers and Children’s Environmental Health (MOCEH) study that included 1751 pregnant women and was performed from 2006 to 2010 in the Republic of Korea. After excluding subjects that were lost to follow-up, 1516 participants were eligible for enrollment in the study. The MOCEH has been previously described in detail [28]. Exposure levels to TRAP were estimated for 1471 women who provided an exact residential address. We excluded 85 children with a gestational age <37 weeks or birth weight <2500 g. Of the remaining 1386 participants, 727 were excluded for the following missing information: AD status at 6 months of age (*n* = 390), maternal body mass index (BMI; *n* = 17), family income (*n* = 36), educational status (*n* = 11), parental allergy history (*n* = 4), parity (*n* = 116), exposure to second-hand smoke (*n* = 1), type of birth (*n* = 149), breastfeeding (*n* = 1), and temperature (*n* = 2). The final study population consisted of 659 pregnant women and their babies. The characteristics of the excluded participants did not differ significantly from those of the study population except for the presence of pets (Table S1). The participants who enrolled in this study provided written informed consent before participation, and the study was approved by the institutional review boards (EGCT 117-4) of Ewha Woman’s University, Dankook University Hospital, and Ulsan University Hospital.

### 2.2. Exposure to TRAP

Exposure to TRAP was assessed based on geocoded residential addresses. The concentrations of PM_10_ and NO_2_ at the participant’s residential address were estimated using land-use regression (LUR) models following a previously described standardized method [29]. In brief, we modeled TRAP concentrations using a regulatory monitoring network and the ambient concentrations of PM_10_ and NO_2_ in the study area. The air pollutants were measured at 37 regulatory monitoring sites of the Korean Ministry of Environment, using centrally and locally available geographic variables as potential predictors. Predictor variables, such as traffic indicators, surrounding land usage, topography, and spatial trends, were derived from a geographic information system (GIS) (ArcGIS version 9.3, ESRI, Redlands, CA, USA). Multiple linear regression models were built using a supervised forward stepwise procedure. The predictor variables that were left in the final LUR model included the length of all roads, traffic intensity on the nearest road, total heavy-duty traffic loads on all roads, and variables representing spatial trends. To specify the uncertainty of air pollution exposure, we calculated the adjusted R^2^, the leave-one-out-cross-validation (LOOCV) R^2^, and the root mean squared error (RMSE) between the predicted and measured concentrations. The model adjusted R^2^ and the LOOCV R^2^ of the NO_2_ models were 0.79 and 0.73, respectively. For PM_10_ models, the adjusted R^2^ and the LOOCV R^2^ were 0.69 and 0.60, respectively. The RMSE was 0.005 ppm for NO_2_ and 3.14 µg/m^3^ for PM_10_, which suggested that the predicted values fitted well with the measured values, as also reported in previous study [30].

The mean PM_10_ and NO_2_ exposure levels were computed by using each participant’s gestational age and birth date. The mean values were first generated for each day, and the daily mean values were then averaged for each pregnant woman over different periods of the pregnancy as follows: 0 to 12 weeks gestation (first trimester), 13 to 28 weeks gestation (second trimester), 29 to 42 weeks gestation (third trimester), and 0 to 42 weeks gestation (entire pregnancy).

### 2.3. Assessment of Green Spaces

To determine green spaces, we used the land cover classification maps (1:25,000) of the Korean Ministry of Environment that detail land cover and use across Korea. These are thematic maps made by using Landsat image data from the National Aeronautics and Space Administration and Korean Arirang satellite images. These spatial information databases depict the surface of the earth based on certain scientific criteria and then color index the area with homogeneous characteristics for display in the form of a map. The maps developed by the Environmental Spatial Information Service Center of the Ministry of Environment are largely divided into urbanized areas including parks, agricultural areas, forest areas, grasslands, wetlands, open areas, and coastal areas. Using the GIS tool (ArcGIS 9.3), we extracted the maps of parks, forest areas, and grasslands. These maps were used to calculate the amount of green space within different buffer sizes (100 m, 200 m, 300 m, and 500 m) around the maternal residential address [27,31].

### 2.4. Health Outcome

Information on doctor-diagnosed infantile AD at the age of 6 months was obtained from the Korean version of the International Study on Allergies and Asthma in Childhood (ISAAC) that included a standardized questionnaire according to an enriched version of the ISAAC standardized protocol. The occurrence of infantile AD was defined as an affirmative answer to the question, “Has a doctor told you that your son/daughter has atopic dermatitis?”.

### 2.5. Statistical Analysis

We used the chi-square test and *t*-test to compare the values and frequencies of the baseline characteristics according to the child’s AD status. Logistic regression models were used to estimate odds ratios (ORs) and 95% confidence intervals (95% CIs) to assess the relationship between AD and TRAP (PM_10_ and NO_2_) or levels of green space. These models were adjusted for the mother’s age (years), body mass index (BMI), gestational age (weeks), parity (0, 1, or ≥2), infant sex (male vs. female), exposure to environmental tobacco smoke (yes vs. no), educational level (more than university vs. less than university), parental history of allergy (yes vs. no), family income (<$2000, $2000–$4000, or >$4000), presence of pets (yes vs. no), residential mobility during pregnancy (yes vs. no), season of birth (winter vs. other seasons), breastfeeding (exclusive breastfeeding vs. exclusive formula or mixed feeding), type of birth (cesarean vs. vaginal), temperature, and humidity. To assess the combined effect of TRAP and green space, we conducted a stratified analysis in which we examined the association between exposure to PM_10_ and NO_2_ during different windows of pregnancy and childhood AD according to the level of green space. We investigated the AD risk resulting from PM_10_ and NO_2_ exposure stratified by tertile of green space. All analyses were performed with Stata 13 (Stata Corporation, College Station, TX, USA). The significance level was set at *p* < 0.05.

## 3. Results

**Figure 1:**
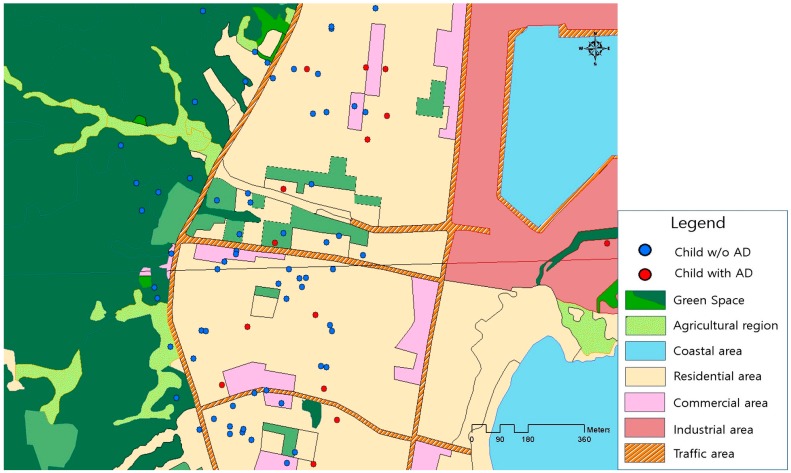
Green space and atopic dermatitis in children. The red filled circles indicate the children with atopic dermatitis; and the blue filled circles indicate the children without atopic dermatitis. The green color indicates green spaces.

Table 1 summarizes the demographic characteristics of the participants with and without AD. Most of the mothers were highly educated (72.7% were university or college graduates), were not exposed to pets (97.4%) during pregnancy, and belonged to a higher socioeconomic status based on an income of ≥$2000/month. Generally, the maternal and infant characteristics different between children with and without AD at 6 months. However, average birth weight and maternal BMI were different between infants with and without AD. Parity and education level were significantly higher among mothers of children with AD. Our study included a fairly large number of AD cases in areas with less green space (Figure 1).

Table 2 summarizes the data for maternal exposure to air pollutants during different periods of pregnancy. The mean exposures during pregnancy were 53.60 μg/m^3^ for PM_10_ and 24.69 ppb for NO_2_. The correlation of the subjects’ trimester-specific PM_10_ and NO_2_ mean concentrations varied across trimesters. The concentration of the first trimester (Pearson’s correlation coefficient, r = 0.51) and the third (r = 0.37) trimester were moderately correlated. Mean concentrations for the whole pregnancy were highly correlated with trimester-specific mean NO_2_ concentration (r = 0.89–0.95) and moderately correlated with mean PM_10_ concentration (r = 0.37–0.73). The mean size of the green space within a 200-m buffer of the residences was 62.00 m^2^. There was a negative correlation between the mean level of green space (200-m buffer) and the PM_10_ and NO_2_ concentrations (r = −0.12 and r = −0.45, respectively; *p* < 0.001).

The association between AD at 6 months of age and exposure to air pollutants in different trimesters is shown in Table 3. We found that the association was significant only for exposure to PM_10_ and NO_2_ during the first trimester of pregnancy. The adjusted OR (95% CI) was 1.219 (1.023–1.452) per 10 μg/m^3^ increase in PM_10_ and 1.353 (1.027–1.782) per 10 ppb increase in NO_2_. Furthermore, an increase in green space area within a 200-m buffer around the home address was associated with a statistically significant decrease in AD risk. The adjusted OR (95% CI) was 0.996 (0.993–0.999). Risk estimates were consistent for a 300 m-buffer size but not significant for the 100-m and 500-m buffer sizes. The unadjusted associations were consistent with the adjusted associations, although somewhat higher ORs were observed for the adjusted associations. The unadjusted association between PM_10_ exposure in the third trimester and AD showed some beneficial effect, but this association remained insignificant in the adjusted model.

We found that the risk of AD during the first trimester was significant at the lower tertiles of green space within the 200-m buffer. The adjusted OR (95% CI) was 1.581 (1.162–2.152) per 10 μg/m^3^ increase in PM_10_ and 1.632 (1.050–2.538) per 10 ppb increase in NO_2_ (Table 4). However, this association disappeared in the upper tertiles. The association between PM_10_ and infantile AD at low levels of green space (tertile 1) for the other buffer sizes (100 m, 300 m, and 500 m) were also significant, but the results were inconsistent for NO_2_ exposure.

## 4. Discussion

The results of this prospective cohort study provide evidence that maternal exposure to PM_10_ and NO_2_ during the first trimester of pregnancy increases infantile AD risk and that this risk is reduced in residential areas with sufficient green space.

Exposure to ambient pollutants during early pregnancy may be important for the development of AD. To date, few studies have reported the effect that TRAP exposure in specific trimesters has on the development of childhood AD [11,13,17]. Three recent cohort studies demonstrated associations between childhood AD and exposure to air pollution by trimester. One study in Taiwan reported that the risk of AD in infants at the age of 6 months was significantly associated with exposure to carbon monoxide, a proxy measure for TRAP, during the first trimester [11]. Another study in Spain found that the risk of AD in children during the first 12–18 months of age was associated with NO_2_ exposure during the second and third trimesters [17]. A third study in China observed that the highest risk of AD after exposure to NO_2_ during the first trimester occurred at 3–6 years of age [13]. Our findings are comparable to those of Huang et al. (2015) [11], who found that exposure to TRAP in the first trimester was a potential risk factor for the development of AD in early childhood. The skin structures of the fetus develop rapidly in the first trimester of pregnancy [32], making it a critical period where the fetus may be highly vulnerable to the harmful effects of air pollution [18,33]. One study suggested that infants may be indirectly affected before birth due to air pollution crossing the protective barrier of the placenta or due to a systemic effect on the health of the mother [34].

We found a stronger influence of TRAP on AD in children residing in areas with less green space, suggesting that AD might be affected by the amount of residential green space. A previous study showed that the amount of forest and agricultural land around homes was inversely associated with the risk of atopic sensitization in children [19], which is associated with the development of AD [35]. Although the exact mechanism of the preventive effect of green space on TRAP-related AD is not known, the environmental biodiversity hypothesis might provide a useful framework for identifying potential underlying mechanisms [36]. Environmental biodiversity has been proposed to contribute to human commensal microbiota, and the microbiota are increasingly understood to influence immune tolerance [37]. Commensal microorganisms on the skin have been shown to correlate with the levels of an inflammatory cytokine that plays a key role in immunologic tolerance (IL-10) [38]. Studies have shown that T-helper 2 chemokines are highly expressed in the skin of patients with AD [39,40] and there have been significant immunologic improvements in children with AD after short-term exposure to forest environments [20]. Maternal exposure to air pollution during pregnancy may alter immune competence in the offspring, thus increasing the risk of AD later in life [41]. Therefore, low green space exposure may exert a suppressive effect on immunologic mechanisms and may, in turn, contribute to the development of TRAP-related AD. However, we could not rule out the possibility of other mechanisms. For example, vegetation may reduce the adverse effect of air pollution on AD by filtering pollutants from the air and lowering ambient temperatures [42,43,44]. Moreover, the helpful effects of vegetation may be outweighed by the adverse effects of pollen [24]. Therefore, in our study, we could not exclude the possible effect of allergens on immunologic improvement.

The main strength of our study was its prospective design beginning in early pregnancy. This design minimized the uncertainty of random error in the covariates of interest. Another strength is the objective estimation of exposure to TRAP at the individual level with temporally adjusted LUR models to capture small-scale variability in pollution. We found an association between increased mean personal exposure to TRAP and the lack of green space surrounding the residential address using individual exposure modeling. However, this method could not estimate personal exposure influenced by maternal time-activity patterns [45]. Higher or lower personal exposures for women at work and away from their residences during pregnancy may have caused exposure misclassification. Also, this study relies on address at recruitment early in pregnancy to generate pollution measures. Those women who moved may have caused additional exposure misclassification mostly for the exposure later in the pregnancy, and this might explain why we find association in the first pregnancy period. Another limitation is that health data were obtained through questionnaires and could have been misclassified. Moreover, cases with missing data were excluded from the study. This may have affected the results due to the loss of information. However, we did not find significant differences between the participants included in this study and those excluded from the study (Table S1).

## 5. Conclusions

Prenatal exposure to TRAP, especially in the first trimester of pregnancy, and low residential green space are both associated with an increased risk of AD at 6 months of age. Exposure to less green space may intensify the effect of PM_10_ and NO_2_ exposure in developing childhood AD. Our findings support the hypothesis that childhood AD may originate in the fetus and may be triggered by TRAP. Our results indicate that strategies to reduce TRAP exposure and to expand green space (forest, parks, etc.) access for pregnant women during early gestation are needed to prevent childhood AD.

## Figures and Tables

**Table 1:** Characteristics of study participants.

Characteristics	Total (*n =* 659)	Atopic Dermatitis at Age 6 Months		*p*-Value ^1^
		Yes (*n =* 168)	No (*n =* 492)	
***Maternal***				
Age (years), *n* (%)				
<30	326 (49.5)	88 (52.7)	238 (48.4)	0.567
30–34	268 (40.7)	65 (38.9)	203 (41.3)	
≥35	65 (9.8)	14 (8.4)	51 (10.3)	
Education, *n* (%)				
<university	180 (27.3)	32 (19.2)	148 (30.1)	0.006
≥university	479 (72.7)	135 (80.8)	344 (69.9)	
Income per month, *n* (%)				
<$2000	177 (26.9)	48 (28.7)	129 (26.2)	0.591
$2000–4000	354 (53.7)	84 (50.3)	270 (54.9)	
>$4000	128 (19.4)	35 (21.0)	93 (18.9)	
Pre-pregnancy BMI ^2^, mean (SD)	21.3 (2.9)	20.9 (3.1)	21.5 (2.8)	0.025
Parity, *n* (%)				
0	315 (47.8)	92 (55.1)	223 (45.3)	0.029
≥1	344 (52.2)	75 (44.9)	269 (54.7)	
History of allergy, *n* (%)				
No	470 (71.3)	113 (67.7)	357 (72.6)	0.227
Yes	189 (28.7)	54 (32.3)	135 (27.4)	
Exposure to SHS, *n* (%)				
No	400 (60.7)	106 (63.5)	294 (59.8)	0.395
Yes	259 (39.3)	61 (36.5)	198 (40.2)	
Residential mobility, *n* (%)				
No	575 (87.2)	140 (83.8)	435 (88.4)	0.125
Yes	84 (12.8)	27 (16.2)	57 (11.6)	
Gestational age (weeks), mean (SD)	39.0 (1.1)	39.1 (1.1)	39.0 (1.1)	0.665
Type of birth, *n* (%)				
Vaginal birth	425 (64.5)	108 (64.7)	317 (64.4)	0.955
Cesarean section	234 (35.5)	59 (35.3)	175 (35.6)	
Season of birth, *n* (%)				
Winter	184 (27.9)	43 (25.8)	141 (28.7)	0.469
Others	475 (72.1)	124 (74.2)	351 (71.3)	
Presence of pets, *n* (%)				
No	642 (97.4)	162 (97.0)	480 (97.6)	0.696
Yes	17 (2.6)	5 (3.0)	12 (2.4)	
***Infant***				
Sex, *n* (%)				
Male	358 (54.3)	89 (53.3)	269 (54.7)	0.757
Female	301 (45.7)	78 (46.7)	223 (45.3)	
Birth weight (grams), mean (SD)	3310 (374.4)	3364 (393.7)	3292 (366.2)	0.030
Breastfeeding, *n* (%)				
Exclusive breast-feeding	238 (36.1)	67 (40.1)	171 (34.8)	0.212
Exclusive formula or mixed feeding	421 (63.9)	100 (59.9)	321 (65.2)	

^1^ Two-sided χ^2^ test and *t*-test where appropriate. ^2^ Weight in kilograms/height in m^2^. Abbreviations: SD, standard deviation; SHS, secondhand smoke; BMI, body mass Index.

**Table 2:** Distributions of prenatal PM_10_/NO_2_ exposure, temperature, humidity, and greenness levels in residential areas of 6-month-old children.

Exposure Variables	Mean (SD)	Minimum	Percentiles			Maximum
			25	50	75	
PM_10_ exposure (μg/m^3^)						
First trimester	54.27 (12.49)	21.47	44.38	53.19	64.11	87.96
Second trimester	52.53 (9.94)	26.70	45.40	51.74	59.84	81.23
Third trimester	54.22 (11.08)	27.31	45.68	54.12	62.50	89.22
Entire pregnancy	53.60 (6.22)	26.04	49.85	53.46	57.49	72.62
NO_2_ exposure (ppb)						
First trimester	24.13 (9.26)	7.37	18.24	22.13	26.66	62.85
Second trimester	24.69 (8.51)	8.40	19.02	22.77	27.70	60.92
Third trimester	25.34 (8.43)	9.05	19.58	23.66	27.85	54.04
Entire pregnancy	24.69 (8.01)	9.42	20.07	22.37	26.49	55.91
Temperature						
First trimester	11.49 (8.62)	−5.82	4.74	9.92	19.5	27.68
Second trimester	11.82 (8.29)	−2.55	4.47	9.99	19.89	25.82
Third trimester	11.59 (8.55)	−4.34	4.54	9.74	19.18	27.01
Entire pregnancy	11.63 (8.28)	−3.74	4.79	9.95	19.68	26.66
Humidity						
First trimester	64.65 (8.64)	46.14	58.24	64.29	71.27	83.38
Second trimester	64.25 (9.11)	39.03	58.64	63.00	70.71	84.95
Third trimester	65.05 (8.13)	49.19	58.58	65.33	71.43	83.21
Entire pregnancy	64.66 (8.31)	44.79	58.76	63.88	70.94	83.26
Green space (m^2^)						
100-m buffer	38.44 (49.40)	0.00	5.73	19.62	47.47	205.38
200-m buffer	62.00 (63.11)	0.00	13.01	38.31	98.49	292.93
300-m buffer	132.52 (89.37)	2.03	52.42	123.36	184.36	440.83
500-m buffer	336.67 (232.79)	2.03	111.83	247.91	538.30	903.54

Abbreviations: SD, standard deviation.

**Table 3:** Risk of atopic dermatitis in 6-month-old children with prenatal exposure to ambient PM_10_/NO_2_ and green space in their area of residence.

Exposure Variable	Atopic Dermatitis	
	Unadjusted	Adjusted ^1^
	OR (95% CI)	OR (95% CI)
PM_10_ (10 μg/m^3^)		
First trimester	1.195 (1.038–1.376) *	1.219 (1.023–1.452) *
Second trimester	1.050 (0.881–1.253)	0.927 (0.725–1.185)
Third trimester	0.834 (0.710–0.980) *	0.975 (0.792–1.201)
Entire pregnancy	1.123 (0.844–1.493)	1.096 (0.795–1.510)
NO_2_ (10 ppb)		
First trimester	1.202 (1.002–1.444) *	1.353 (1.027–1.782) *
Second trimester	1.045 (0.852–1.282)	1.115 (0.839–1.480)
Third trimester	0.999 (0.811–1.230)	1.254 (0.934–1.684)
Entire pregnancy	1.105 (0.891–1.369)	1.269 (0.936–1.721)
Residential surrounding green space		
100-m buffer	0.993 (0.993–1.001)	0.997 (0.993–1.001)
200-m buffer	0.997 (0.994–0.999) *	0.996 (0.993–0.999) *
300-m buffer	0.999 (0.996–0.999) *	0.997 (0.995–0.999) *
500-m buffer	0.999 (0.999–1.000)	0.999 (0.998–1.000)

Abbreviations: OR, odds ratios; CI, confidence interval; ppb, part per billion. ^1^ Adjusted for maternal age, education, income, body mass index, history of allergy, exposure to secondhand smoke, residential mobility, gestational age, the presence of pets, infant sex, birth weight, season of birth, breastfeeding, mode of delivery, temperature, and humidity. * Significant at *p* < 0.05.

**Table 4:** Risk of atopic dermatitis in 6-month-old children with exposure to ambient PM_10_/NO_2_ during the first trimester of pregnancy stratified by the level of green space in the residential area.

Green Space in Residential Area (m^2^) ^1^	*n*	Atopic Dermatitis			
		PM_10_ during 1st Trimester (10 μg/m^3^)		NO_2_ during 1st Trimester (10 ppb)	
		Unadjusted OR (95% CI)	Adjusted ^2^ OR (95% CI)	Unadjusted OR (95% CI)	Adjusted ^2^ OR (95% CI)
100-m buffer					
Tertile 1 (<11.1)	220	1.289 (1.025–1.621) *	1.454 (1.079–1.959) *	1.087 (0.828–1.426)	1.453 (0.944–2.236)
Tertile 2 (11.1–28.8)	221	1.055 (0.821–1.356)	1.047 (0.739–1.482)	1.189 (0.805–1.756)	0.838 (0.420–1.675)
Tertile 3 (>28.8)	218	1.174 (0.901–1.530)	0.938 (0.639–1.378)	1.202 (0.752–1.921)	1.281 (0.643–2.551)
200-m buffer					
Tertile 1 (<21.1)	220	1.284 (1.018–1.619) *	1.581 (1.162–2.152) **	1.169 (0.887–1.541)	1.632 (1.050–2.538) *
Tertile 2 (21.1–64.2)	220	1.174 (0.911–1.512)	1.120 (0.813–1.542)	1.060 (0.726–1.546)	1.044 (0.568–1.919)
Tertile 3 (>64.2)	219	1.058 (0.814–1.375)	0.852 (0.575–1.261)	1.284 (0.710–2.323)	1.063 (0.468–2.411)
300-m buffer					
Tertile 1 (<63.3)	220	1.268 (1.002–1.604) *	1.455 (1.063–1.993) *	1.140 (0.872–1.491)	1.506 (0.973–2.330)
Tertile 2 (63.3–171.5)	220	1.042 (0.815–1.330)	1.183 (0.858–1.631)	0.935 (0.618–1.413)	1.020 (0.538–1.934)
Tertile 3 (>171.5)	219	1.196 (0.910–1.572)	0.995 (0.679–1.458)	1.411 (0.833–2.391)	1.599 (0.723–3.537)
500-m buffer					
Tertile 1 (<158.5)	220	1.356 (1.068–1.721) *	1.451 (1.076–1.957) *	1.171 (0.892–1.537)	1.330 (0.877–2.017)
Tertile 2 (158.5–444.8)	220	1.107 (0.847–1.446)	1.246 (0.961–1.803)	0.998 (0.643–1.550)	1.066 (0.563–2.020)
Tertile 3 (>444.8)	219	1.076 (0.836–1.385)	1.005 (0.716–1.409)	1.434 (0.911–2.225)	1.882 (0.857–4.133)

Abbreviations: OR, odds ratios; CI, confidence interval; ppb part per billion. ^1^ The individual green space size within the buffer area. The distribution of green space was divided into tertiles. ^2^ Adjusted for maternal age, education, income, body mass index, history of allergy, exposure to secondhand smoke, residential mobility, gestational age, the presence of pets, infant sex, birth weight, season of birth, breastfeeding, mode of delivery, temperature, and humidity. * Significant at *p* < 0.05, ** *p* < 0.01.

## Supplementary Materials

The following are available online at www.mdpi.com/1660-4601/15/1/102/s1, Table S1: Characteristics of study participants who were included or not included in the present study.

## References

[1] Weidinger S., Novak N. (2016). Atopic dermatitis. Lancet.

[2] Williams H., Flohr C. (2006). How epidemiology has challenged 3 prevailing concepts about atopic dermatitis. J. Allergy Clin. Immunol..

[3] Sturgill S., Bernard L.A. (2004). Atopic dermatitis update. Curr. Opin. Pediatr..

[4] Ahn K., Kim J., Kwon H., Chae Y., Hahm M.L., Lee K.J., Park Y.M., Lee S.M., Han M., Kim W.K. (2011). The prevalence of symptoms of asthma, allergic rhinoconjunctivitis, and eczema in Korean children: Nationwide cross-sectional survey using complex sampling design. J. Korean Med. Assoc..

[5] Ricci G., Patrizi A., Baldi E., Menna G., Tabanelli M., Masi M. (2006). Long term follow-up of atopic dermatitis: Retrospective analysis of related risk factors and association with concomitant allergic diseases. J. Am. Acad. Dermatol..

[6] Wang I.J., Guo Y.L., Weng H.J., Hsieh W.S., Chuang Y.L., Lin S.J., Chen P.C. (2007). Environmental risk factors for early infantile atopic dermatitis. Pediatr. Allergy Immunol..

[7] Hasunuma H., Ishimaru Y., Yoda Y., Shima M. (2014). Decline of ambient air pollution levels due to measures to control automobile emissions and effects on the prevalence of respiratory and allergic disorders among children in Japan. Environ. Res..

[8] Song S., Lee K., Lee Y.M., Lee J.H., Lee S.I., Yu S.D., Peak D. (2011). Acute health effects of urban fine and ultrafine particles on children with atopic dermatitis. Environ. Res..

[9] Deng Q., Lu C., Ou C., Chen L., Yuan H. (2016). Preconceptional, prenatal and postnatal exposure to outdoor and indoor environmental factors on allergic diseases/symptoms in preschool children. Chemosphere.

[10] Deng Q., Lu C., Li Y., Sundell J., Norbäck D. (2016). Exposure to outdoor air pollution during trimesters of pregnancy and childhood asthma, allergic rhinitis, and eczema. Environ. Res..

[11] Huang C.C., Wen H.J., Chen P.C., Chiang T.L., Lin S.J., Guo Y.L. (2015). Prenatal air pollutant exposure and occurrence of atopic dermatitis. Br. J. Dermatol..

[12] Liu W., Cai J., Huang C., Hu Y., Fu Q., Zou Z., Sun C., Shen L., Wang X., Pan J. (2016). Associations of gestational and early life exposures to ambient air pollution with childhood atopic eczema in Shanghai, China. Sci. Total Environ..

[13] Lu C., Deng L., Ou C., Yuan H., Chen X., Deng Q. (2017). Preconceptional and perinatal exposure to traffic-related air pollution and eczema in preschool children. J. Dermatol. Sci..

[14] Miyake Y., Tanaka K., Fujiwara H., Mitani Y., Ikemi H., Sasaki S., Ohya Y., Hirota Y. (2010). Residential proximity to main roads during pregnancy and the risk of allergic disorders in Japanese infants: The Osaka Maternal and Child Health Study. Pediatr. Allergy Immunol..

[15] Sbihi H., Allen R.W., Becker A., Brook J.R., Mandhane P., Scott J.A., Sears M.R., Subbarao P., Takaro T.K., Turverym S.W. (2015). Perinatal exposure to traffic-related air pollution and atopy at 1 year of age in a multi-center Canadian birth cohort study. Environ. Health Perspect..

[16] Wen H.J., Chen P.C., Chiang T.L., Lin S.J., Chuang Y.L., Guo Y.L. (2009). Predicting risk for early infantile atopic dermatitis by hereditary and environmental factors. Br. J. Dermatol..

[17] Aguilera I., Pedersen M., Garcia-Esteban R., Ballester F., Basterrechea M., Esplugues A., Fernadez-Somoano A., Lertxundi A., Tardon A., Sunyer J. (2013). Early-life exposure to outdoor air pollution and respiratory health, ear infections, and eczema in infants from the INMA study. Environ. Health Perspect..

[18] Jedrychowski W., Perera F., Maugeri U., Mrozek-Budzyn D., Miller R.L., Flak E., Morz E., Jacek R., Spenler J.D. (2011). Effects of prenatal and perinatal exposure to fine air pollutants and maternal fish consumption on the occurrence of infantile eczema. Int. Arch. Allergy Immunol..

[19] Ruokolainen L., von Hertzen L., Fyhrquist N. (2015). Green areas around homes reduces atopic sensitization in children. Allergy.

[20] Seo S.C., Park S.J., Park C.W., Yoon W.S., Choung J.T., Yoo Y. (2015). Clinical and immunological effects of a forest trip in children with asthma and atopic dermatitis. Iran. J. Allergy Asthma Immunol..

[21] Tamosiunas A., Grazuleviciene R., Luksiene D., Dedele A., Reklaitiene R., Baceviciene M., Vencloviene J., Bernotiene G., Radisauskas R., Malinauskiene V. (2014). Accessibility and use of urban green spaces, and cardiovascular health: Findings from a Kaunas cohort study. Environ. Health.

[22] Sugiyama T., Leslie E., Giles-Corti B., Owen N. (2008). Associations of neighborhood greenness with physical and mental health: Do walking, social coherence and local social interaction explain the relationships?. J. Epidemiol. Community Health.

[23] Andrusaityte S., Grazuleviciene R., Kudzyte J., Bernotiene A., Dedele A., Nieuwenhuijsen M.J. (2016). Associations between neighbourhood greenness and asthma in preschool children in Kaunas, Lithuania: A case-control study. BMJ Open.

[24] Lovasi G.S., O’Neil-Dunne J.P., Lu J.W., Sheehan D., Perzanowski M.S., Macfaden S.W., King K.L., Matte T., Miller R.L., Hoepner L.A. (2013). Urban tree canopy and asthma, wheeze, rhinitis, and allergic sensitization to tree pollen in a New York City birth cohort. Environ. Health Perspect..

[25] Pilat M.A., McFarland A., Snelgrove A., Collins K., Waliczek T.M., Zajicek J. (2012). The effect of tree cover and vegetation on incidence of childhood asthma in metropolitan statistical areas of Texas. HortTechnology.

[26] Dadvand P., Villanueva C.M., Font-Ribera L., Martinez D., Basagaña X., Belmonte J., Vrihheid M., Grazulevicieene R., Kogevinas M., Nieuwenhuijsen M.J. (2014). Risks and benefits of green spaces for children: A cross-sectional study of associations with sedentary behavior, obesity, asthma, and allergy. Environ. Health Perspect..

[27] Grazuleviciene R., Danileviciute A., Dedele A., Vencloviene J., Andrusaityte S., Uždanaviciute I., Nieuwenhuijsen M.J. (2015). Surrounding greenness, proximity to city parks and pregnancy outcomes in Kaunas cohort study. Int. J. Hyg. Environ. Health.

[28] Kim B.M., Ha M., Park H.S., Lee B.E., Kim Y.J., Hong Y.C., Kim Y., Chang N., Roh Y.-M., Kim B.-N. (2009). The Mothers and Children’s Environmental Health (MOCEH) study. Eur. J. Epidemiol..

[29] Lee J.Y., Leem J.H., Kim H.C., Hwang S.S., Jung D.Y., Park M.S., Kim J.-A., Lee J.-J., Park N.-W., Kang S.-C. (2012). Land Use Regression Model for Assessing Exposure and Impacts of Air Pollutants in School Children. J. Korean Soc. Atmos. Environ..

[30] Lamichhane D.K., Kim H.C., Choi C.M., Shin M.H., Shim Y.M., Leem J.H., Ryu J.S., Nam H.S., Park S.M. (2017). Lung Cancer Risk and Residential Exposure to Air Pollution: A Korean Population-Based Case-Control Study. Yonsei Med. J..

[31] Schüle S.A., Gabriel K.M.A., Bolte G. (2017). Relationship between neighbourhood socioeconomic position and neighbourhood public green space availability: An environmental inequality analysis in a large German city applying generalized linear models. Int. J. Hyg. Environ. Health.

[32] Loomis C.A., Koss T., Chu D., Bolognia J.L., Jorizzo J.L., Rapini R.P. (2008). Embryology. Dermatology.

[33] Bieber T. (2008). Atopic dermatitis. N. Engl. J. Med..

[34] Currie J., Neidell M. (2005). Air pollution and infant health: What can we learn from California’s recent experience?. Q. J. Econ..

[35] Jøhnke H., Norberg L.A., Vach W., Høst A., Andersen K.E. (2006). Patterns of sensitization in infants and its relation to atopic dermatitis. Pediatr. Allergy Immunol..

[36] Kuo M. (2015). How might contact with nature promote human health? Promising mechanisms and a possible central pathway. Front. Psychol..

[37] Rook G.A. (2013). Regulation of the immune system by biodiversity from the natural environment: An ecosystem service essential to health. Proc. Natl. Acad. Sci. USA.

[38] Hanski I., von Hertzen L., Fyhrquist N., Koskinen K., Torppa K., Laatikainen T., Karisola P., Auvinen P., Paulin l., Makela M.J. (2012). Environmental biodiversity, human microbiota, and allergy are interrelated. Proc. Natl. Acad. Sci. USA.

[39] Fujisawa T., Nagao M., Hiraguchi Y., Katsumata H., Nishimori H., Iguchi K., Kato Y., Higasiura M., Ogawauchi I., Tamaki K. (2009). Serum measurement of thymus and activation-regulated chemokine/CCL17 in children with atopic dermatitis: Elevated normal levels in infancy and age-specific analysis in atopic dermatitis. Pediatr. Allergy Immunol..

[40] Furusyo N., Takeoka H., Toyoda K., Murata M., Maeda S., Ohnishi H., Fukiwake N., Uchi H., Furue M., Hayashi J. (2007). Thymus and activation regulated chemokines in children with atopic dermatitis: Kyushu University Ishigaki Atopic Dermatitis Study (KIDS). Eur. J. Dermatol..

[41] Baïz N., Slama R., Béné M.C., Charles M.A., Kolopp-Sarda M.N., Magnan A., Thiebaugeorges O., Faure G., Annesi-Maesano I. (2011). Maternal exposure to air pollution before and during pregnancy related to changes in newborn’s cord blood lymphocyte subpopulations. The EDEN study cohort. BMC Pregnancy Childbirth.

[42] Dadvand P., de Nazelle A., Triguero-Mas M., Schembari A., Cirach M., Amoly E., Figueras F., Basagana X., Ostro B., Nieuwenhuijsen M. (2012). Surrounding greenness and exposure to air pollution during pregnancy: An analysis of personal monitoring data. Environ. Health Perspect..

[43] Nowak D.J., Crane D.E., Stevens J.C. (2006). Air pollution removal by urban trees and shrubs in the United States. Urban For. Urban Green..

[44] Paoletti E., Bardelli T., Giovannini G., Pecchioli L. (2011). Air quality impact of an urban park over time. Procedia Environ. Sci..

[45] Weir C.H., Yeatts K.B., Sarnat J.A., Vizuete W., Salo P.M., Jaramillo R., Cohn R.D., Chu H., Zeldin D.C., London S.J. (2013). Nitrogen dioxide and allergic sensitization in the 2005–2006 national health and nutrition examination survey. Respir. Med..

