# Supplementary material for: Preventive Effect of Residential Green Space on Infantile Atopic Dermatitis Associated with Prenatal Air Pollution Exposure
Source: Int J Environ Res Public Health. 2018 Jan 9;15(1):102. doi: 10.3390/ijerph15010102 (PMC5800201; doi:10.3390/ijerph15010102)
Supplement: Supplementary file 1 [file ijerph-15-00102-s001.pdf]

Table S1. Characteristics of study participants who were included and not included in the present study.

Characteristics	Included participants (n = 659)	Excluded participants (n = 727)	p-value [1]
Maternal			
Age (years), mean (SD)	30.5 (3.5)	30.2 (3.8)	0.161
Education, n (%)			
< university	180 (27.3)	167 (23.0)	0.062
≥ university	479 (72.7)	560 (77.0)	
Income per month, n (%)			
< $ 2000	177 (26.9)	168 (27.4)	0.967
$ 2000-4000	354 (53.7)	325 (53.0)	
> $ 4000	128 (19.4)	120 (19.6)	
Pre-pregnancy BMI [2], mean (SD)	21.3 (2.9)	21.0 (2.9)	0.083
Parity, n (%)			
0	315 (47.8)	260 (52.4)	0.297
≥ 1	344 (52.2)	236 (47.6)	
History of allergy, n (%)			
No	470 (71.3)	225 (67.0)	0.287
Yes	189 (28.7)	111 (33.0)	
Exposure to SHS, n (%)			
No	400 (60.7)	397 (54.6)	0.109
Yes	259 (39.3)	330 (45.4)	
Residential mobility, n (%)			
No	575 (87.2)	301 (89.3)	0.344
Yes	84 (12.8)	36 (10.7)	
Gestational age (weeks), mean (SD)	39.0 (1.1)	39.1 (1.05)	0.295
Type of birth, n (%)			
Vaginal birth	425 (64.5)	385 (63.4)	0.436
Cesarean section	234 (35.5)	222 (36.6)	
Season of birth, n (%)			
Winter	184 (27.9)	227 (31.2)	0.179
Others	475 (72.1)	500 (68.8)	
Presence of pets, n (%)			
No	642 (97.4)	321 (94.7)	0.026
Yes	17 (2.6)	18 (5.3)	
Infant			
Sex, n (%)			
Male	358 (54.3)	364 (50.1)	0.102
Female	301 (45.7)	363 (49.9)	
Birth weight (grams), mean (SD)	3310 (374.4)	3307 (378.8)	0.866
Breastfeeding, n (%)			
Exclusive breast-feeding	238 (36.1)	138 (41.0)	0.136
Exclusive formula or mixed feeding	421 (63.9)	199 (59.0)	
Atopic dermatitis at 6 months, n (%)			
No	492 (74.7)	232 (68.8)	0.051
Yes	167 (25.3)	105 (31.2)	

Of the 812 participants who were excluded, we considered 727 after removing 85 participants for low birth weight and preterm birth. [1] P value by *t* test or χ^2 test. [2] Weight in kilograms/height in m^2.
